# Optical orientation of nematic liquid crystal droplets via photoisomerization of an azodendrimer dopant

**DOI:** 10.3762/bjnano.9.81

**Published:** 2018-03-13

**Authors:** Sergey A Shvetsov, Alexander V Emelyanenko, Natalia I Boiko, Alexander S Zolot'ko, Yan-Song Zhang, Jui-Hsiang Liu, Alexei R Khokhlov

**Affiliations:** 1 M.V. Lomonosov Moscow State University, Moscow, 119991, Russia; 2 P.N. Lebedev Physical Institute, Moscow, 119991, Russia; 3 National Cheng Kung University, Tainan, 70101, Taiwan

**Keywords:** dendrimer, droplets, nematic liquid crystal, orientational transition, photo-orientation

## Abstract

Two sequential transformations of the orientational structure in nematic liquid crystal droplets containing a dendrimer additive (nanosized macromolecules with light-absorbing azobenzene terminal moieties) under light irradiation in the UV–blue spectral range were investigated. The origin of these transitions is in the change of the boundary conditions due to photoisomerization of the dendrimer adsorbed onto the liquid crystal–glycerol interface. It was shown that the photoisomerization processes of dendrimer molecules in a liquid crystal are accompanied by a spatial rearrangement of their azobenzene moieties, which is the key point in the explanation of the observed effects.

## Introduction

Azobenzene compounds represent a very convenient tool for the development of photosensitive materials [1–4]. This feature is associated with the capability of these molecules to change their shape due to absorption of light. A stable *trans* isomer can be excited by a light quantum and transformed into the metastable *cis* form, and vice versa, the *cis* isomer after light excitation returns into *trans* form. The anisotropic interaction of azobenzene molecules with each other, as well as with their environment, essentially depends on their shape (rod-like for *trans* isomers and having no distinct anisometry for *cis* isomers). This leads to the modification of physical properties of different kinds of soft matter, such as light-driven polymers [5–6], elastomers [7–9] microgel particles [10–11], micelles [12–13], nematic liquid crystals (NLCs) [7,14], liquid-crystalline (LC) polymers [15–18] and Langmuir–Blodgett films [19–20] with light-controllable supramolecular structures.

Among the variety of different materials containing azobenzene derivatives, there is special interest in host–guest systems consisting of a mesophase matrix and a small concentration (*<*1 wt %) of dopant. In particular, they reveal higher optical nonlinear response [14,21]. Some kinds of the polymer dopants, such as a dendrimer with azobenzene terminal moieties [22–23] or a comb-shaped polymer with H-bonded side-chain azobenzene fragments [24–25], can induce the homogeneous orientation of NLC films. These polymer additives are usually adsorbed onto the cell substrates and provide homeotropic anchoring of the NLC film. The boundary conditions can be changed to planar and then return to homeotropic again by photoisomerization processes. The effects of NLC film orientation are very similar to the bulk mediated photoalignment [26–27], which are influenced by exchange of the dopant between the surface and the bulk.

**Figure 1:**
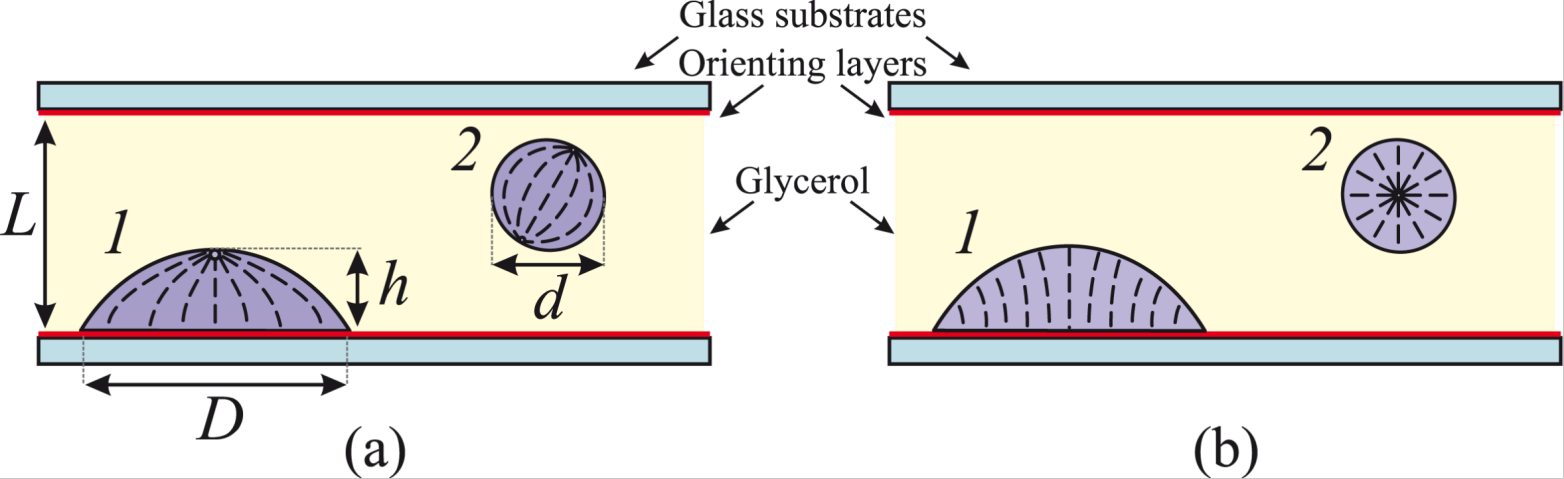
Sketch of a glass cell with NLC droplets embedded into glycerol with planar (a) and homeotropic (b) boundary conditions. The NLC director distribution in the droplet resting on the solid substrate (*1*) and in the spherical droplet in the bulk of glycerol (*2*) are shown by dashed lines. Adapted from [30], with the permission of AIP Publishing.

Recently, it was shown that NLC photo-orientation due to the azobenzene dopant photoisomerization can also occur at the interface between nematic and isotropic liquid [28]. It is known [29] that NLC microdroplets in the bulk of glycerol usually have bipolar orientational structure with two boojum defects (Figure 1a). As shown in [28], an azodendrimer additive incorporated into NLC droplets initiates the formation of homeotropic boundary conditions, at which the director orientation of the NLC droplets is radial (Figure 1b). It was assumed that the molecular branches of the adsorbed dendrimer molecules are mostly oriented perpendicularly to the NLC–glycerol interface and align the NLC molecules in the same direction. The UV irradiation causes the *trans*→*cis* isomerization of the dopant, the *cis* isomers of the terminal moieties provide the planar alignment of NLC.

In our experiment reported as communication in [30], the NLC was doped with a carbosilane dendrimer of the fifth generation. The obtained results appeared to be different from the ones mentioned above. The presence of the azodendrimer additive used in the experiment did not influence the orientational structure of NLC droplets, i.e., with or without dopant the NLC director distribution of microdroplets was bipolar. However, under near-UV light illumination, the bipolar orientation of NLC droplets changed to a radial orientation.

In addition, the photoinduced change in the NLC director distribution was observed in the droplets resting on the solid substrate [30]. The NLC director distribution with boojum defect on the top of the droplet (Figure 1a) can be reversibly changed to almost homeotropic NLC alignment (Figure 1b). The interest in these geometries of NLC droplets is explained by their better stability in comparison with the droplets in the bulk of the solvent. Consequently, they are more promising for some applications such as the detection of different chemical compounds [31–33].

In this paper, we present new experimental data for NLC droplets in glycerol showing a sequence of reversible photoinduced orientational transitions caused by the isomerization of dendrimer dopant under light illumination with different wavelengths. Both NLC droplets in the bulk of glycerol and in contact with the solid substrate are considered. To understand the nature of these transitions, the spatial configuration of dendrimer molecules in the nematic matrix is considered. We elaborate a method for estimation of the orientational order parameter of the azobenzene fragments in a nematic matrix at different percentages of isomers.

## Results and Discussion

### Orientational structure modulation in NLC droplets

#### NLC droplets in the bulk of glycerol

**Figure 2:**
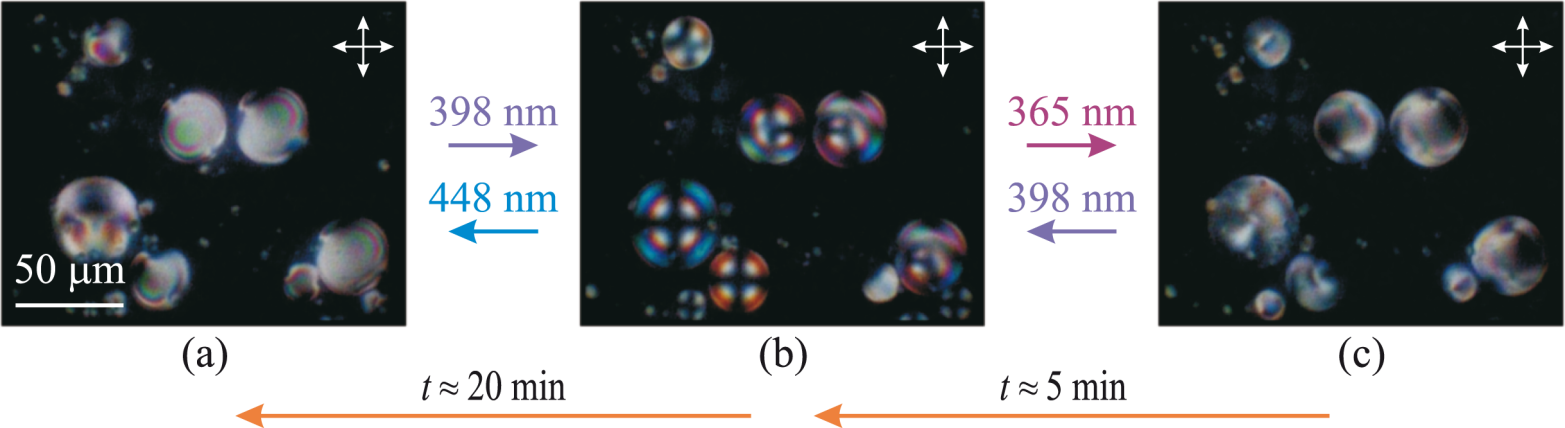
POM images of NLC droplet structures in the bulk of glycerol obtained at light-emitting diode irradiation with wavelengths of (a) 448 nm, (b) 398 nm and (c) 365 nm. The arrows show the direction of transitions. The double arrows show the directions of the crossed polarizers.

Structural transformations were investigated for the droplets of NLC doped with the azobenzene dendrimer (G5) in glycerol environment. The microdroplets (with diameter *d* ≈ 30 μm) of NLC in the bulk of glycerol have the same bipolar structure with two point defects on the droplet surface as the undoped NLC (Figure 2a). The director distribution of NLC droplets is determined by planar surface conditions in glycerol. Thus, the presence of polymer additive does not disturb the orientational structure of the droplets in the absence of light-emitting diode (LED) illumination.

The LED illumination with λ_max_ = 398 nm causes a bipolar-to-radial orientational transition in NLC droplets due to the change in the boundary conditions from planar to homeotropic (Figure 2a,b). The same orientational structures were formed under light of λ_max_ = 406 nm. The subsequent light illumination with λ_max_ = 448 nm (or 422 nm, or 466 nm) restores the initial bipolar structure (Figure 2a). These structure transformations were first described in our communication [30].

After the light irradiation with λ_max_ = 398 nm, the irradiation with light of λ_max_ = 365 nm (as well as of λ_max_ = 384 nm) tends to turn the radial structure of the droplets to the one (Figure 2c) that is close to the initial bipolar structure (Figure 2a). The NLC droplet orientation is caused by planar anchoring conditions or anchoring conditions tilted at a small angle [34–35].

It is possible to induce the orientational transitions in the opposite direction. For instance, the NLC structures shown in Figure 2c transform into those presented in Figure 2b under light irradiation with λ_max_ = 398 nm (or 406 nm), while the structures shown in Figure 2b transform into the ones presented in Figure 2a under light irradiation with λ_max_ = 448 nm (or 466 nm). In each case, the formation time of stable orientational structures in NLC droplets varies from several to several tens of seconds depending on the droplet size.

The orientational structures obtained by light irradiation can spontaneously transform into the initial state in the absence of LED illumination. The bipolar droplet structure shown in Figure 2c first relaxes to radial (Figure 2b) over a time of approximately 5 min and then, over a time of 20 min, the droplets return back to a bipolar structure (Figure 2a).

Thus, the NLC droplets can consequently change their configuration twice when the wavelength of the irradiation light is decreased. These transformations of NLC droplets strongly depending on the light wavelength can be explained by changing concentrations of *trans* and *cis* isomers of the dendrimer azobenzene moieties.

#### NLC droplets in contact with the solid substrate

**Figure 3:**
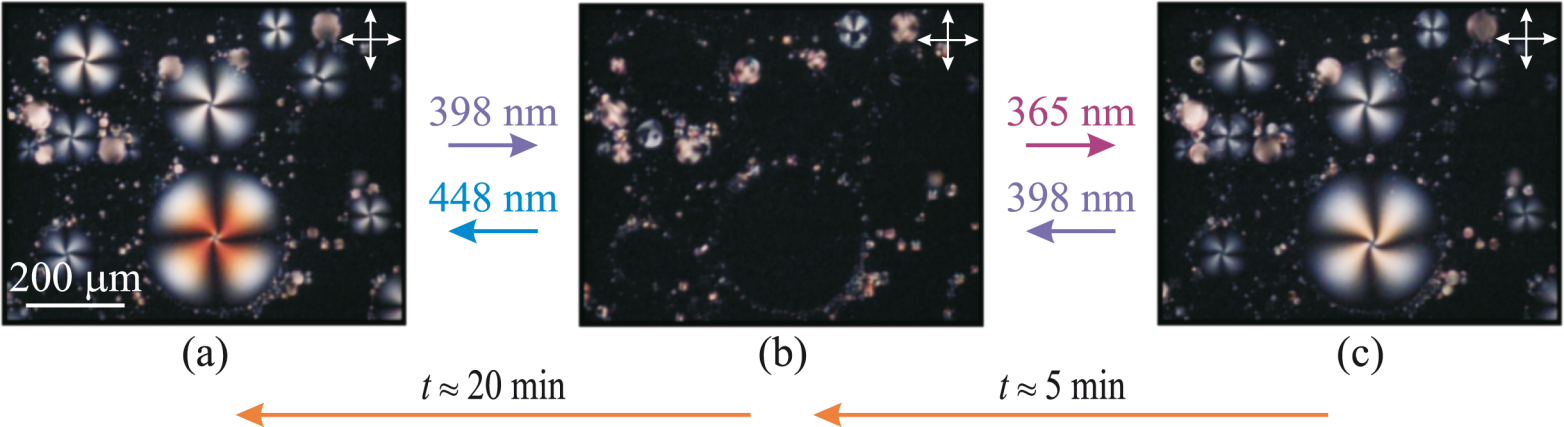
POM images of NLC droplet structures in contact with the solid substrate obtained under light-emitting diode irradiation with wavelengths of (a) 448 nm, (b) 398 nm and (c) 365 nm. The arrows show the direction of transitions. The double arrows show the directions of the crossed polarizers.

Let us consider the orientational transitions of NLC droplets resting on the cell substrate. Before any LED irradiation, the NLC droplets exhibit homeotropic alignment on the substrate coated with an orienting compound and planar alignment on the glycerol interface. This leads to the director distribution with one bujoom defect on the top (Figure 1a). These droplets are visualized in crossed polarizers as slightly twisted Maltese crosses, while the smaller droplets between them are the previously described droplets in the bulk of glycerol (Figure 3a). A twist can be explained by the difference between elastic splay and twist constants (*K*_11_ and *K*_22_) [36] or/and a contribution of the elastic constant *K*_24_ [37] to the energy. The breadth of droplets *D* varies from 15 to 250 μm. By measuring the droplet size before and after adsorption, we evaluated the average ratio of a droplet height *h* to its length *D*, which equals 0.2.

The LED illumination causes the same changes in the boundary conditions of the NLC droplets with glycerol as in the case of spherical NLC droplets in the bulk of glycerol described above. Namely, the influence of light of λ_max_ = 398 nm (or 406 nm) leads to homeotropic boundary conditions with glycerol (Figure 1b), and the droplet structure becomes almost homogeneous with the director being preferably oriented normally to the substrate and visualized as dark regions (Figure 3b). The UV illumination with λ_max_ = 365 nm (or 384 nm) provides the boojum defect formation due to planar (or close to planar) anchoring of NLC with glycerol (Figure 3c). The reverse changes from planar to homeotropic anchoring of NLC with glycerol can be produced by light irradiation with λ_max_ = 398 nm (or 406 nm), and then, from homeotropic to degenerated planar anchoring, by light irradiation with λ_max_ = 448 nm (or 422 nm, or 466 nm). Note that the photoinduced transitions between the initial, with one boojum, and the structure free from point defects (Figure 3a,b) were first obtained in [30].

Orientational transitions in NLC droplets resting on the substrate can also occur spontaneously in the same manner, as in the droplets in the bulk. After 365 nm illumination, the orientational structure with one bujoom (see Figure 1a and Figure 3c) becomes almost homeotropic over a time of about 5 min (see Figure 1b and Figure 3b) and finally, over a time of 20 min, the bujoom defect is formed again (see Figure 1a and Figure 3a).

### Interpretation of photoinduced orientational transitions

Summarizing the obtained results for the orientational transitions in spherical NLC droplets and in the droplets in contact with the solid substrate, we can conclude that the change of the boundary conditions is associated with the variation in isomer concentrations of dendrimer terminal moieties. To clarify the influence of these concentrations on the NLC–glycerol boundary conditions, we need to consider the spatial configuration of the G5 dendrimer.

It is known [38] that the terminal moieties with flexible spacers can pack loosely with respect to each other without coiling. The existence of smectic and columnar mesophases of pure G5 dendrimer, as well as large absorption dichroism [39] of G5 dendrimer dissolved in NLC, clearly shows that the dendrimer branches can be oriented in some direction due to molecular interaction. In contrast, the formation of bend *cis* isomers significantly reduces the excluded volume of the macromolecule and disturbs the arrangement of the terminal moieties.

**Figure 4:**
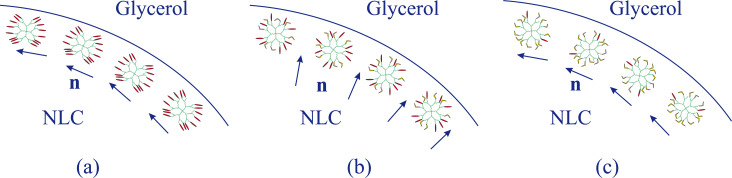
Schematic illustration of dendrimer molecules near the NLC–glycerol interface and of the NLC director **n** (a) when all azo moieties of the dendrimer are in *trans* state, (b) when the concentration of *trans* isomers is comparable to the concentration of *cis* isomers, (c) when almost all isomers exist in the *cis* state.

Considering NLC droplets, we can suggest that a layer of the adsorbed dendrimer macromolecules at the NLC–glycerol interface works as a command surface [26]. When a significant amount of isomers exists in the rod-like *trans* state, they are oriented along the NLC director **n** and do not influence the NLC orientation in contact with glycerol. The orientation remains degenerated planar as without dendrimer (Figure 4a). Note that the dendrimer moieties do not penetrate into glycerol due to their hydrophobic properties.

Under light irradiation, the azobenzene fragments partially transform into the bent *cis* isomers having a very small order parameter in the nematic matrix [40]. In the case of azobenzene monomers (M), the order parameter of the *cis* isomers, *S*_cis_, equals ca. 0.1, which is much smaller than the order parameter of the nematic matrix. In our case, *S*_cis_ should be further reduced by the disturbance of dendrimer molecular branches, and thus, can be neglected.

In the environment of disordered *cis* isomers, the *trans* isomers are oriented radially with respect to the dendrimer molecule center (Figure 4b). On the macroscopic level, the order parameter of the *trans* isomers should be zero. The NLC molecules tend to be oriented along the nearest *trans* isomers of dendrimer molecules forming a layer at the interface. As a result, the NLC director is aligned homeotropically at the interface. A similar situation, in which NLC molecules are ordered in the vicinity of nanosized particles, was considered in [41]. However, if the most of azo moieties are converted into the *cis* isomer under UV irradiation, they provide a degenerated planar orientation of NLC at the interface (Figure 4c).

It is also possible to explain the orientational structure transitions obtained in NLC microdroplets containing azodendrimers of the third generation considered in [28] in the same terms. If the molecular branches of the dendrimers are much shorter than in our case, before irradiation, the *trans* isomers can be ordered along the radius of each dendrimer molecule and cause the homeotropic anchoring at the NLC–glycerol interface. Under UV irradiation, almost all azofragments are converted to *cis* isomers, which provide the degenerated planar orientation. This situation is similar to the transition observed in our case (Figure 2b,c).

### Influence of photoisomerization on the order parameter of the *trans* isomer

To prove the described concept, we have clarified experimentally how the formation of *cis* isomers affects the order parameter of *trans* isomers of the G5 dendrimer and also of the azobenzene monomer (M), which is similar to the G5 terminal moieties, incorporated into the nematic matrix. To this end, we used the spectroscopic method that is described in the Experimental section.

**Figure 5:**
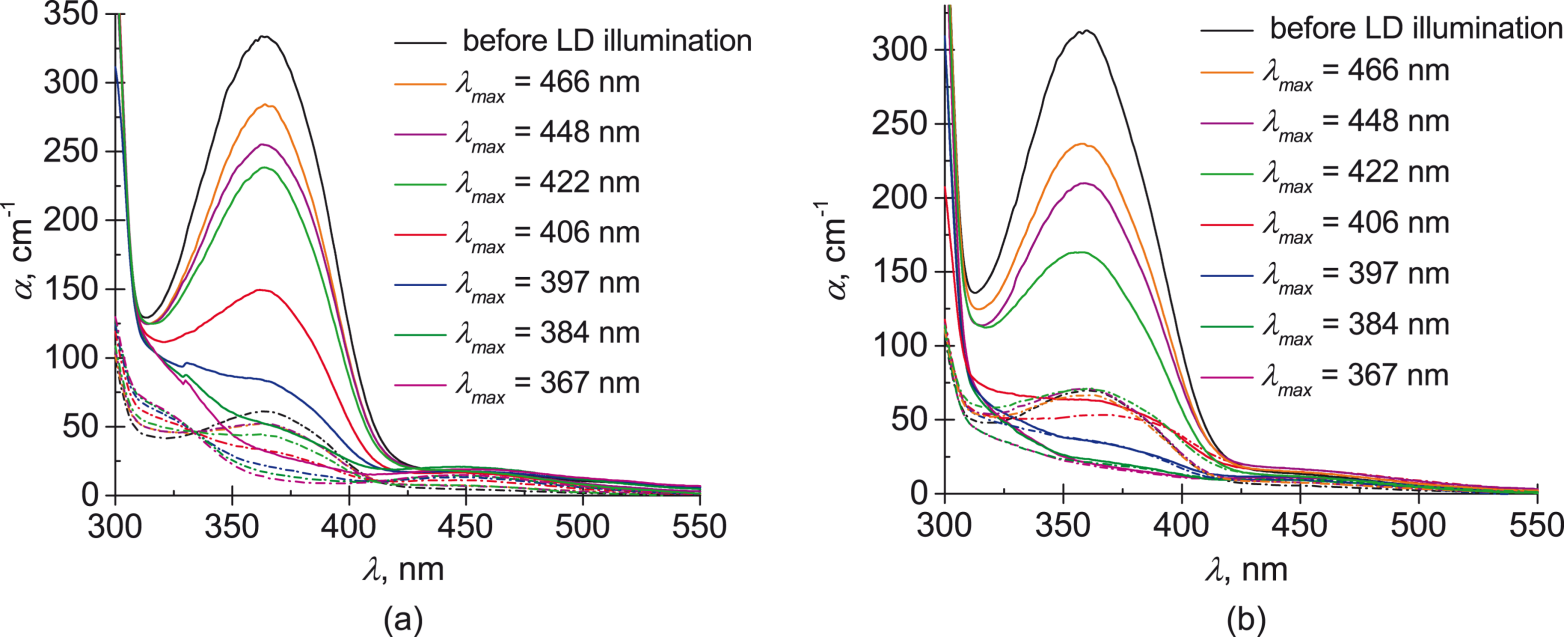
Absorption spectra of the extraordinary (solid curves) and ordinary (dashed curves) waves for (a) NLC doped with monomer M and (b) NLC doped with dendrimer G5 before and after LED illumination with λ_max_ = 365, 384, 398, 405, 422, 448 and 466 nm.

The polarized absorption spectra of planar cells with NLC doped with M or G5 were measured before and after LED irradiation (Figure 5). The decrease of the light wavelength leads to a sequential reduction of the absorption near the absorption band of the *trans* isomer (about 365 nm). At the same time, the dichroism *D* = (α_e_ − α_o_)/(α_e_ + α_o_) of the samples is also reduced. The reduction of *D* is larger for the G5-doped NLC than for the M-doped NLC. For example, after LED illumination with λ_max_ = 406 nm, the dichroism *D* is equal to 0.8 for M-doped NLC and 0.1 for G5-doped NLC. This difference can be explained by the higher concentration of *cis* isomers or by the reduction of the *trans* isomer order parameter, *S*_trans_.

**Figure 6:**
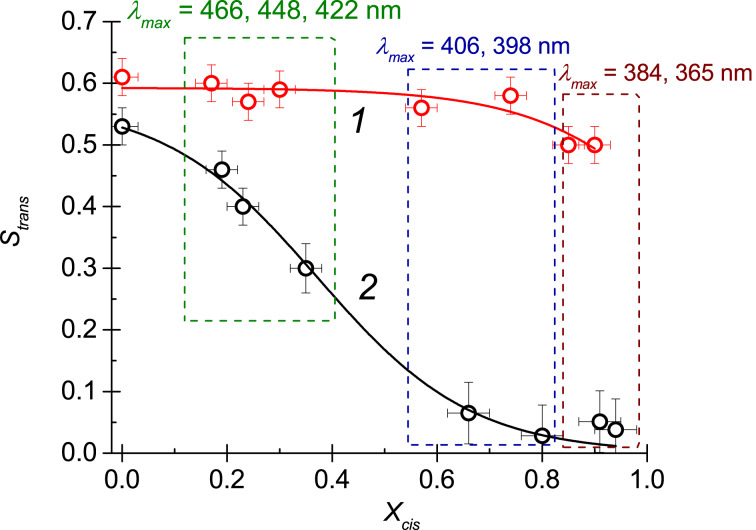
The *trans* isomer order parameter, *S*_trans_, as a function of the relative concentration of *cis* isomers, *X*_cis_, for (1) NLC doped with monomer M and (2) NLC doped with dendrimer G5.

The obtained data allow one to estimate the *trans* isomer order parameter and the ratio of isomers under varying LED irradiation. First, the *cis* isomer absorption coefficient should be determined. It is convenient to use the spectra after LED illumination with λ_max_ = 365 and 398 nm due to relatively large absorption coefficients and difference in spectral dichroism. Using the procedure described in the Experimental section, for the NLC doped with monomer M, we have found the relative concentrations of *cis* isomers *X*_cis_ = 0.90 at λ_max_ = 365 nm and *X*_cis_ = 0.74 at λ_max_ = 398 nm, while the absorption coefficient at λ = 365 nm was found to be α_cis_ = 6 cm^−1^. For the G5-doped NLC, we have found *X*_cis_ = 0.93 at λ_max_ = 365 nm, *X*_cis_ = 0.81 at λ_max_ = 398 nm, and α_cis_ = 10 cm^−1^ at λ = 365 nm. Then, we obtain the dependence of the *trans* isomer order parameter, *S*_trans_, on the *cis* isomer concentration, *X*_cis_, induced by the other light sources (Figure 6).

The change in light wavelength λ_max_ from blue to UV causes a monotonous increase of *cis* isomer concentration of both azobenzene compounds M and G5. From Figure 6 it follows that, in the case of monomer dopant M (Figure 6, upper red curve), the *trans* isomer order parameter, *S*_trans_, almost remains unchanged with the variation in *cis* isomer concentration, *X*_cis_. Since the *S*_trans_ is supposed to be close to the NLC order parameter [40], we can conclude that the formation of *cis* isomers does not affect the order parameter of the nematic matrix up to *X*_cis_ ≈ 0.8. A small reduction of *S*_trans_ at *X*_cis_ *>* 0.8 generally means that the nematic order parameters of *trans* isomers and nematic matrix can be influenced directly by the presence of *cis* isomers to a very small extent. On the contrary, for the NLC doped with dendrimer G5, the monotonous increase of *X*_cis_ is accompanied by a dramatic decrease of *S*_trans_ (Figure 6, lower black curve). This reduction is not associated with the change in NLC order parameter and can be explained by a rearrangement of *trans* isomers surrounded by disordered *cis* isomers.

Let us now return to the description of photoinduced transitions in the NLC droplets with dendrimer additive. The obtained data for *S*_trans_, under LED irradiation match the proposed concept of light-induced effects in NLC droplets (described in the section above). Under LED irradiation with λ_max_ = 422, 448 or 466 nm (or without any LED irradiation), *S*_trans_ is relatively large (*S*_trans_ ≥ 0.3 at *X*_cis_ *<* 0.4), and the dendrimer azobenzene moieties are oriented mostly along the NLC director. Thus, the dendrimer additive does not influence the degenerated planar NLC orientation (Figure 2a and Figure 3a). Under LED irradiation with λ_max_ = 398 or 406 nm, a significant amount of *cis* isomers is formed. This leads to a reduction of *trans* isomer order parameter (*S*_trans_ *<* 0.3 at 0.4 *< X*_cis_ *<* 0.8), and the NLC director becomes oriented normally to the NLC–glycerol interface (Figure 2b and Figure 3b). Finally, when almost all azo moieties are in the *cis* state (*S*_trans_ *<* 0.1 at *X*_cis_ *>* 0.8) under illumination with λ_max_ = 384 or 465 nm, the orientation of NLC director becomes planar (Figure 2c and Figure 3c).

If the NLC is doped with azobenzene monomers (M), the *trans* isomers are always oriented along the NLC molecules and unable to change the director orientation, which was proved experimentally [30], where it was shown that the orientational structure does not change under LED illumination in NLC droplets containing the low-molar-mass azobenzene additives. It should be mentioned that this statement is correct only at relatively small concentrations of azobenzene monomer, when the dopant barely influences the NLC ordering. If the amount of azo compound is large, the change in the isomer concentration can greatly affect the properties of the NLC system. For instance, radial–bipolar structure transitions occur in microdroplets of azoxybenzene NLC under UV irradiation [42–43].

## Conclusion

The orientational structure transitions in NLC droplets associated with the change in the boundary conditions have been studied. An influence of light on the orientational structures of NLC droplets is achieved by photoisomerization of azobenzene dendrimer G5 doped into the NLC and spontaneously adsorbed onto the NLC–glycerol interface.

The NLC droplets in the bulk of glycerol and those resting on the solid substrate were considered. In both cases, it was shown that a sequence of the orientational transitions can be induced by light irradiation with wavelengths decreasing from blue to UV. The NLC boundary conditions at the interface with glycerol change first from planar to homeotropic and then again to planar. This nontrivial behavior is explained in terms of the variation of the *trans* isomer order parameter of azobenzene dendrimer moieties located near the NLC–glycerol interface. When most of the azobenzene dendrimer terminal moieties are in the *trans* state, they tend to be oriented along the NLC director, and the droplet boundary conditions remain degenerated planar. If a number of azo moieties is converted to the *cis* state, they rearrange the neighboring *trans* isomers, which tend to orient the NLC director normally to the NLC surface. Finally, when the concentration of *trans* isomers dramatically reduces, the NLC anchoring returns to degenerated planar.

## Experimental

### NLC droplet samples and experimental technique

The nematic host NLC-6816 (Merck) consisting of cyclohexane derivatives, and thus transparent in the UV spectral range, was doped with 0.1 wt % of dendrimer of the fifth generation (G5). This compound consists of a nanosized carbosilane dendritic matrix with 128 terminal azobenzene mesogenic groups covalently linked to the dendrimer periphery by flexible aliphatic chains [30]; the synthesis of G5 is described in [44].

Next, the NLC was mixed with glycerol and filled into a glass cell (Figure 1). The cell consisted of two glass plates separated by Teflon spacers with the thickness *L* = 100 μm. The inner substrates of the cell were coated with an orienting compound (chromium stearyl chloride), providing a strong homeotropic anchoring of the NLC. The substrate treatment leads to a certain orientation of the NLC droplets which rest on the cell substrate.

To induce the photoisomerization processes, we used a set of light emitting diodes (LEDs) with maximum emissions at λ_max_ = 365, 384, 398, 406, 422, 448 and 466 nm and FWHM = 10, 13, 19, 16, 16, 25 and 22 nm, respectively. The LED illumination was normal to the cell plane; the average light intensity (measured by a Hioki 3664 power meter) was *I* = 3–6 mW/cm^2^, depending on the particular LED, and exposure time was τ_exp_ = 60 s. The unpolarized light fell normally to the sample.

The NLC droplet structures were analyzed by polarized-light optical microscopy (POM). The induced orientational structures in the NLC droplets were stable at least for several tens of seconds, which allowed us to observe them in the microscope (Carl Zeiss Axio-Pol) with crossed polarizers just after illumination. All experiments were carried out at room temperature.

### NLC film samples for spectral measurements

To measure the *trans* isomer order parameter as a function of the isomer concentrations, NLC-6816 doped with 0.1 wt % of G5 dendrimer and NLC-6816 doped with 0.1 wt % of monomer M [39], which is identical to the dendrimer terminal moieties, were investigated. These mixtures were placed into 100 μm-thick quartz cells. A planar orientation of the NLCs was reached by coating the cell surfaces with polyimide and rubbing in a particular direction. The comparison of these two samples allows one to reveal the role of binding of azobenzene moieties in the dendrimer molecules.

To induce the photoisomerization processes in NLC films, the same LEDs as above were used. The exposure time (τ_exp_ = 60 s) and light intensity (*I* = 3–6 mW/cm^2^) were sufficiently large to achieve a saturation of isomer concentration, i.e., the absorption spectra do not change at a further increase of τ_exp_ and *I*.

It should be noted that the LED irradiation does not cause any orientational effects in the NLC films. The effects of light-induced director reorientation in the films of NLCs doped with M or G5 occur at light intensities which are three or even four orders of magnitude higher [45].

The polarized absorption spectra were recorded with the help of an MC-122 spectrometer (Proscan Special Instruments). The absorption of samples does not change (at least this variation is not larger than the measurement accuracy) after LED illumination over a time of several minutes due to the long *cis* isomer life-time (ca. 12 h), so that the spectra were measured immediately after LED illumination.

### Determination of *X*_cis_ and *S*_trans_

Let us consider the dynamics of *trans*
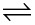
*cis* photoisomerization processes of azobenzene compounds. First, for simplicity, the influence of light on an isotropic solution of azobenzene compound will be analyzed. The relative change of *X*_cis_ (the ratio between *cis* isomer concentration *N*_cis_ and total concentration of isomers *N*_Σ_) can be described by the following equation [46]:

[1]
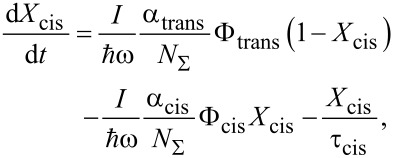


where *I* and ω are the intensity and frequency of the irradiating (pump) light; α_trans_, α_cis_ are the absorption coefficients when all isomers are in *trans* or *cis* states, respectively; τ_cis_ is the time of spontaneous *cis*→*trans* relaxation. The first term on the right-hand side of Equation 1 describes the increase of the *cis* isomer fraction due to the light excitation of *trans* isomers and their possible change (with the probability Φ_trans_) of the conformational state. The second and third terms describe the decrease of *cis* isomer fraction due to the light excitation (with the probability Φ_cis_ = 1 − Φ_trans_) and thermal *cis*→*trans* relaxation.

The thermal relaxation can be neglected at 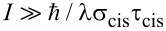. Using this condition, in the stationary case (d*X*_cis_/d*t* = 0), Equation 1 can be rewritten in the following form:

[2]
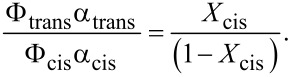


Thus the relative concentrations of *trans* and *cis* isomers are determined by the quantum yields (Φ_trans_ and Φ_cis_) and absorption coefficients and do not depend on exposure time and light intensity.

In the case of an anisotropic solution, the light absorption depends on the direction of light propagation. In contrast to disordered *cis* isomers (we neglect their order parameter, i.e., *S*_cis_ = 0), the *trans* isomers tend to be oriented along the NLC director and have different absorption coefficients 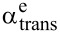 and 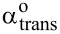 for extraordinary and ordinary light waves. These absorption coefficients can be expressed in terms of the *trans* isomer order parameter, *S*_trans_, [47] as

[3]
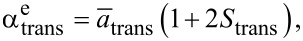


[4]
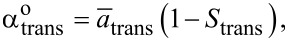


where 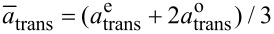 is the average absorption coefficient of the *trans* isomers.

If the unpolarized light beam falls normally on the planarly oriented NLC film, the absorption coefficient equals

[5]
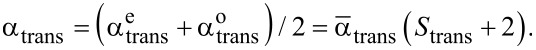


Without light irradiation, there are only *trans* isomers. Therefore the absorption spectrum of *trans* isomers corresponds to the spectrum obtained in a probe beam in the absence of pump light. To find the spectrum of *cis* isomers, one can use an approach described in [48], where the light illumination with two different wavelengths (λ*_i_*, *i* = 1, 2) is considered. In this case, using Equation 2 and Equation 5, we obtain the following equation:

[6]
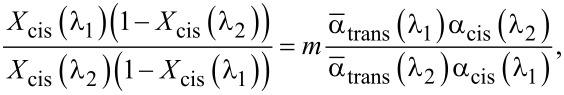


where 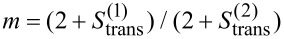, 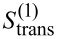 and 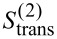 are the *trans* isomer order parameters at fractions of *cis* isomers *X*_cis_(λ_1_) and *X*_cis_(λ_2_). The quantity *m* − 1 is approximately 0.5Δ*S*, which is less than 0.05 at 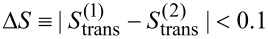. For further consideration, one can assume that *m* = 1. Note that in our case a variation of the parameter *m* in Equation 6 slightly influences the determination of α_cis_ and gives an error of about 2%.

Experimentally, one can measure the absorption coefficients 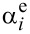 and 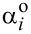 of extraordinary and ordinary probe waves, respectively, under pump light irradiation. These coefficients are expressed through the absorption coefficients and the relative concentrations of *trans* and *cis* isomers as

[7]
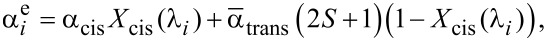


[8]
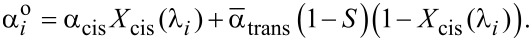


The average absorption coefficient 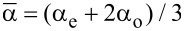 is equal to:

[9]
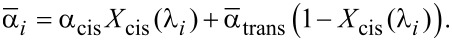


From Equation 9 one can find the following ratio between *X*_cis_(λ_1_) and *X*_cis_(λ_2_) using the average absorption coefficients 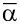 at an arbitrary wavelength λ:

[10]
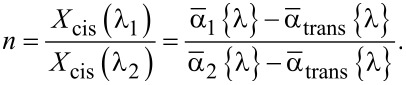


It is convenient to calculate ratio *n* for the wavelength λ corresponding to the absorption peak, to maximize the change in measured absorption with respect to the experimental error. Equation 6 and Equation 10 allow one to find the relative isomer concentrations *X*_cis_(λ_1_) and *X*_cis_(λ_2_):

[11]
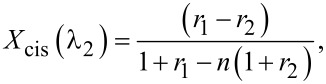


[12]
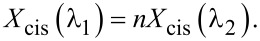


where *r_i_* = (α*_i_* − α_trans_)/α_trans_ is the relative change in absorption measured at the wavelength of the pump light, λ*_i_*, while the ratio *n*, as mentioned above, still should be better calculated at the wavelength corresponding to the absorption maximum.

Then the absorption coefficient of *cis* isomers α_cis_ at arbitrary wavelengths λ can be found from Equation 9:

[13]
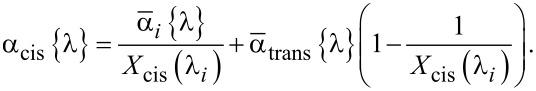


Equation 7 and Equation 8 allow one to find *S*_trans_ and *X*_cis_ under light irradiation with arbitrary λ:

[14]
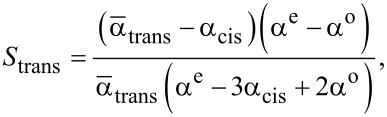


[15]
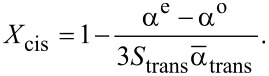


From Equation 14 and Equation 15 one can find the dependence of *S*_trans_ on *X*_cis_ under light irradiation with different wavelengths.

Thus, we have elaborated the method to determine the relative concentrations of isomers and the *trans* isomer order parameter. Summarizing this method, we need to use two light sources (at λ*_i_*, *i* = 1, 2) to find the absorption spectrum α_cis_ and relative concentrations of *cis* isomers *X*_cis_(λ*_i_*). Then, as α_cis_ is known, we can determine *X*_cis_ and *S*_trans_ for the light illumination with other wavelengths λ.
